# Symptomatic Pancreaticojejunal Anastomotic Stricture After Pancreaticoduodenectomy Managed by Longitudinal Pancreaticogastrostomy: A Report of Two Cases

**DOI:** 10.7759/cureus.23884

**Published:** 2022-04-06

**Authors:** Mridul Pansari, Michael Jacobs, Sachin Patil, Sushruta Nagarkatti

**Affiliations:** 1 Hepato-Pancreato-Biliary (HPB) Surgery, Ascension Providence Hospital, Southfield, USA

**Keywords:** hematochezia, pancreaticojejunal anastomotic stricture, pancreaticogastrostomy, revision of pj anastomosis, whipple procedure, pj stricture, hpb surgery

## Abstract

Symptomatic pancreaticojejunal anastomotic stricture is a rare complication following pancreaticoduodenectomy. Literature for the management of pancreaticojejunal anastomotic strictures is limited. Revision of pancreaticojejunostomy anastomosis, endoscopic dilation, stenting of pancreaticojejunal stricture, and modified Puestow procedure have all been described with variable outcomes. We present a report of two patients who developed symptomatic pancreaticojejunal anastomotic stricture following a pancreaticoduodenectomy, managed by longitudinal pancreaticogastrostomy with no complications, and resolution of symptoms with an average follow-up interval of 45 months.

## Introduction

The incidence, presentation, and management of symptomatic pancreaticojejunal anastomotic strictures are infrequently reported in the literature. This is attributed to the fact that most of the pancreaticoduodenectomies were being performed in patients with malignancies, with a median survival of 20 months and five-year survival of 15% [1,2,3]. As more and more pancreaticoduodenectomies are being performed for benign and pre-malignant conditions and with the improvement in life expectancy of patients with malignancies, more long-term complications have been unveiled. This includes pancreaticojejunal anastomotic stricture. There is limited data on the management strategies and their success rates. Most of the case reports and series favor surgical revision of the original pancreaticojejunal anastomosis [4]. Revision of pancreaticojejunal anastomoses can be a very challenging operation. Longitudinal pancreaticogastrostomy has been described in only a few case reports as a possible option with good outcomes. We present here two cases of symptomatic pancreaticojejunal anastomotic stricture after pancreaticoduodenectomy which were managed with longitudinal pancreaticogastrostomy.

## Case presentation

Case 1

**Figure 1:**
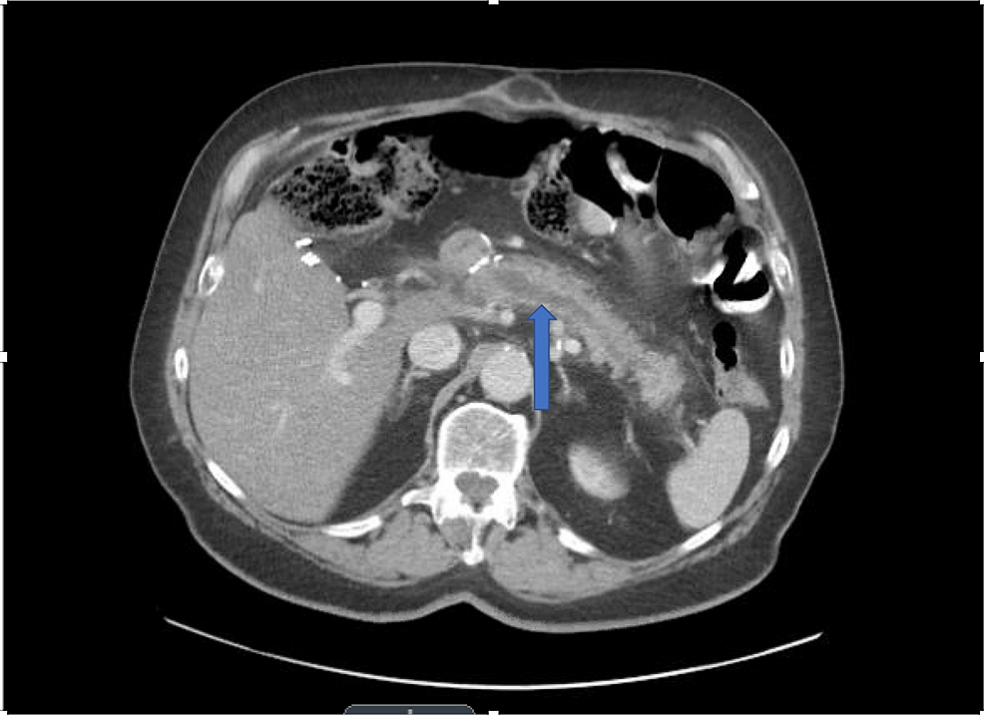
Dilation of the main pancreatic duct to 1 cm secondary to pancreaticojejunostomy stricture

**Figure 2:**
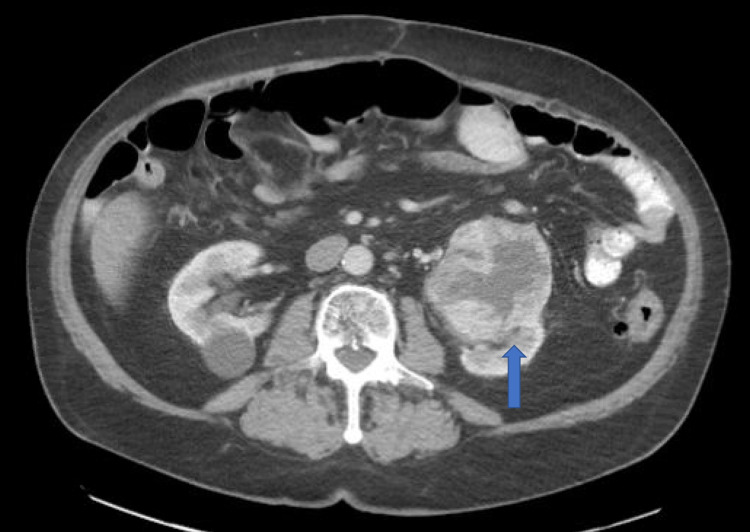
Heterogeneously enhancing mass in left kidney

An 80-year-old male presented with hematochezia and was found to have a non-obstructing 2 cm fungating circumferential mass in the ascending colon during a colonoscopy. The patient was also found to have a 3.6 cm solid enhancing pancreatic head mass without biliary or pancreatic ductal dilation and no vessel involvement. An endoscopic ultrasound-guided fine-needle aspiration (EUS/FNA) of the pancreatic head mass was positive for pancreatic ductal adenocarcinoma. The biopsy of the colonic mass was consistent with colon adenocarcinoma. There was no evidence of metastatic disease on staging CT. The patient underwent an open right hemicolectomy and a Whipple operation in February 2017. The pancreas was noted to be soft 2-3/10 (10 being fibrotic pancreas) with a small duct. Pancreaticojejunostomy was performed in two layers with 4-0 Maxon used for duct to mucosa anastomosis and 3-0 silk for the anterior and posterior layers of pancreatic parenchyma to the seromuscular lining of jejunal limb anastomosis. An 8 Fr pediatric feeding tube was placed across the anastomosis. Pathologic staging was T1N0M0 for the colon cancer and T3N1M0 for the pancreatic cancer. The patient recovered uneventfully. The patient underwent adjuvant chemotherapy (Xeloda/Gemzar) and refused radiation. In November 2018, the patient was admitted for acute pancreatitis, which was managed conservatively. CT abdomen/pelvis at that time showed pancreatic duct dilation to be 6-7 mm. The patient was admitted with another episode of pancreatitis in February 2019 at a hospital in a different state. He was readmitted in April 2019 with another episode of pancreatitis. Imaging at this time showed pancreatic ductal dilation to 1 cm (Figure 1). During this admission, the patient was taken for a longitudinal pancreaticogastrostomy after the pancreatitis resolved. A longitudinal pancreaticogastrostomy was performed over 1 inch using 2-0 Maxon. The patient also underwent a left radical nephrectomy for a known, suspicious, increasing in size, left renal mass (Figure 2), which turned out to be renal cell carcinoma, during the same operation. The patient recovered well from the operation.

Case 2

**Figure 3:**
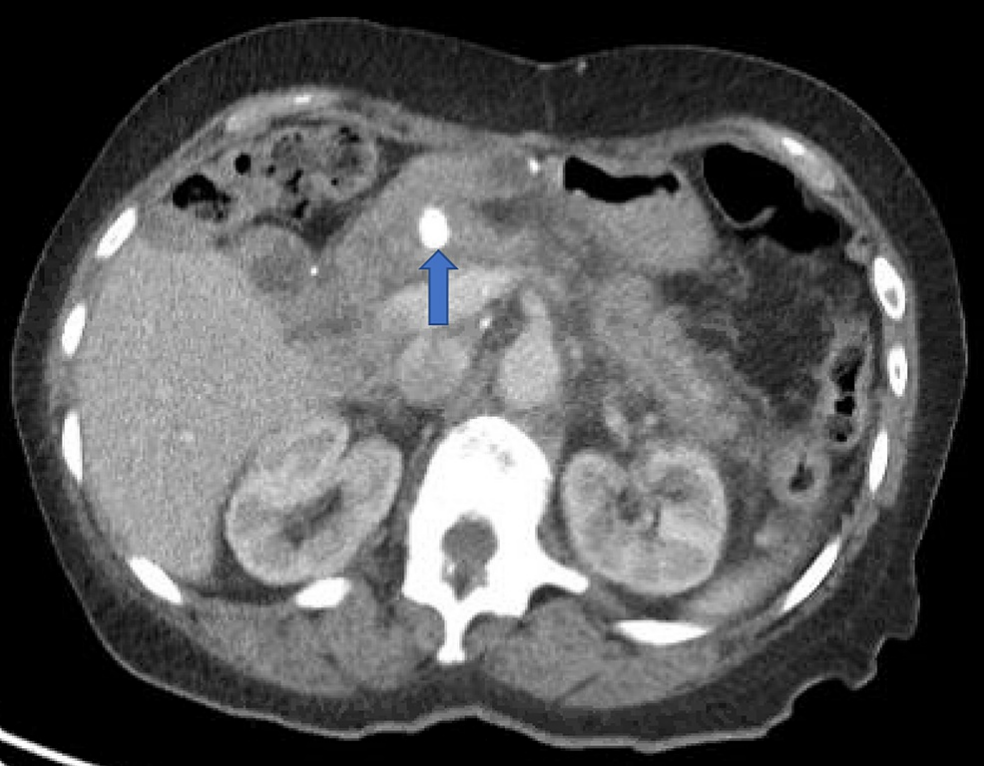
Pancreaticolith

**Figure 4:**
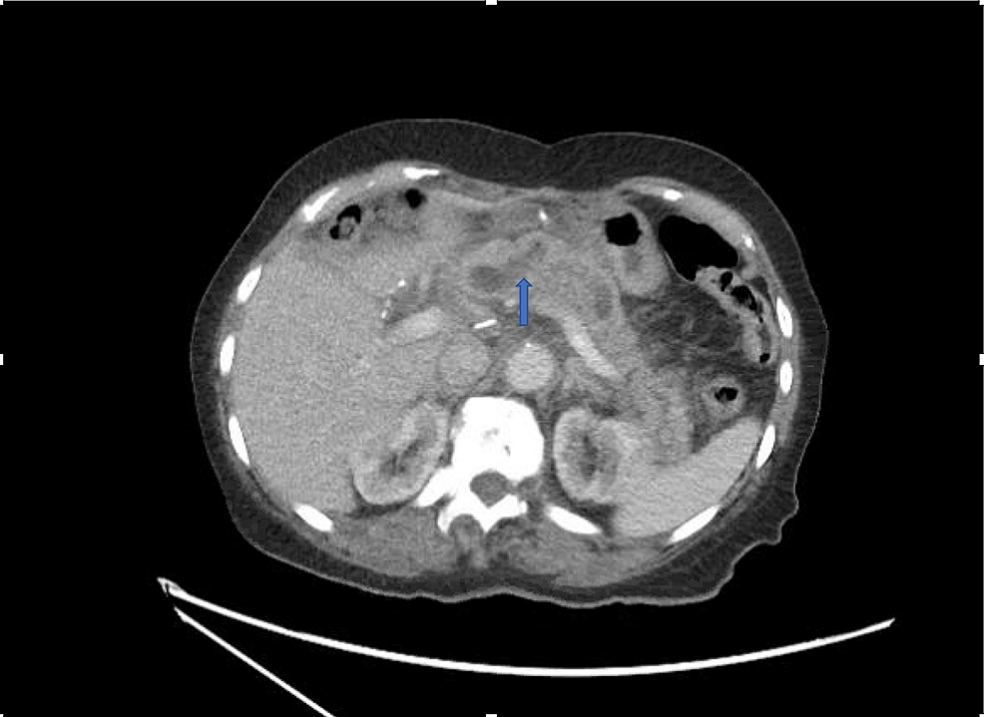
Dilated pancreatic duct secondary to pancreaticojejunostomy anastomosis stricture

The second case is an 82-year-old female who had a Whipple operation for a periampullary tubulovillous adenoma in May 2011. The pancreas was noted to be soft 3-4/10 (10 being fibrotic pancreas). The pancreatic duct was noted to be 3-4 mm in size. Pancreaticojejunostomy was performed in two layers with 4-0 Maxon used for duct to mucosa anastomosis and 3-0 silk for the anterior and posterior layers of pancreatic parenchyma to the seromuscular lining of jejunal limb anastomosis. In October 2018, the patient was admitted with worsening mid-epigastric abdominal pain and identified to have a 1.4 cm obstructing pancreaticolith (Figure 3) in the pancreatic head with distal dilation of the pancreatic duct (Figure 4). The patient underwent longitudinal pancreaticogastrostomy and pancreatic lithotomy. The pancreaticogastrostomy was performed using 2-0 Maxon sutures. The patient recovered uneventfully and was discharged.

Both patients have remained symptom-free since their last surgery and were recently seen at follow-up at 44 and 46 months, respectively.

## Discussion

Pancreaticoduodenectomy is now commonly performed. Indications range from benign conditions such as chronic pancreatitis, to premalignant conditions like intraductal papillary mucinous neoplasm, to malignancy. The 30-day mortality has reduced to less than 5%, particularly in high-volume centers. The morbidity on the other hand has remained in the 30% range [1,2,3]. Immediate complications following pancreaticoduodenectomy include clinically relevant pancreatic fistula (seen in approximately 15% of patients), delayed gastric emptying, hemorrhage, and various infections. Intermediate-term problems include endocrine and exocrine insufficiency [1,2,3]. Less is understood regarding long-term complications such as anastomotic strictures.

This is explained by the fact that historically the majority of pancreaticoduodenectomies were performed in patients with pancreatic head malignancy and the life expectancy was not sufficiently long to unmask this complication. With technical advances in surgery, the performance of pancreatic resections at high-volume centers, and an increasing proportion of surgery for benign conditions, there is now more robust long-term data available on larger cohorts of long-term survivors after pancreaticoduodenectomy.

The reported incidence of pancreaticojejunal anastomotic stricture is 2-11% with most series reporting incidence between 2% and 4% [5,6]. The average time of presentation ranges from 12 to 46 months [4,5]. Clinical presentation can be late-onset chronic abdominal pain or recurrent pancreatitis episodes after pancreaticoduodenectomy. Patients who had postoperative pancreatic fistula after pancreatoduodenectomy are more likely to develop pancreaticojejunal stricture; however, this is controversial. The diagnosis leading to pancreaticoduodenectomy, gland texture at the time of pancreaticoduodenectomy, and pancreaticojejunostomy anastomotic technique were not associated with pancreaticojejunostomy stricture [5]. The symptoms usually trigger an investigation, which is usually a pancreatic protocol computed tomography scan or magnetic resonance cholangiopancreatography with or without secretin enhancement [5]. The characteristic imaging finding is stricture at the level of the pancreaticojejunal anastomosis and upstream pancreatic ductal dilation in the pancreatic remnant. It should be noted here that these imaging findings may be present in asymptomatic patients and do not warrant any intervention.

The endoscopic treatment is more successful with the use of a rendezvous technique but requires technical expertise and long-term outcome data is not available. Most of the studies showed that the results of endoscopic interventions are not promising [7,8]. In a recent study by Cioffi et al., 60% of their patients underwent endoscopic ultrasound-guided intervention but none benefitted from it. Cioffi et al reported morbidity of 26% after surgical revision of pancreaticojejunal anastomotic stricture. No postoperative mortality occurred. Of the patients, 78% experienced resolution of symptoms without recurrent acute pancreatitis after pancreaticojejunal revision during a median follow-up of 30 months. Durable symptom resolution was reported among 60% of patients with chronic pancreatitis. In a study described by Demirjian et al., four out of seven patients with pancreaticojejunal anastomotic stricture were managed initially with endoscopic retrograde cholangiopancreatography (ERCP) [5]. All of those patients required revision of the original pancreaticojejunal anastomosis. Two patients underwent modified Puestow’s procedure. The pancreaticojejunal stricture of one patient was completely inaccessible due to dense adhesions. At the median follow-up time of 18 months, there was no mortality but four out of seven patients were completely pain-free. Although these results are promising, these surgeries were performed by experienced surgeons at a high-volume center and may not be applicable widely. Alternative options such as longitudinal pancreaticogastrostomy, their technical safety, and clinical efficacy need to be continued to be explored.

## Conclusions

Longitudinal pancreaticogastrostomy remains an attractive option in patients with symptomatic pancreaticojejunal anastomotic stricture. It provides a theoretical advantage in terms of favorable anatomy and fewer adhesions. Moreover, a pancreaticogastric anastomosis is accessible via endoscopy for any intervention that may be needed in the long run. We need more case reports/series and long-term outcome data after pancreaticogastrostomy to make definitive conclusions.
